# Quantitative Systems Pharmacology Models of Anti‐Amyloid Treatments for Alzheimer’s Disease: A Systematic Review

**DOI:** 10.1002/alz70859_102996

**Published:** 2025-12-25

**Authors:** Lara Herriott, Mark Coles, Eamonn Gaffney, Nicolas Fournier, Nicolas Sanchez, Olivier Sol, Marija Vukicevic, Andrea Pfeifer, Anke Post, Jonathan Wagg

**Affiliations:** ^1^ University of Oxford, Oxford United Kingdom; ^2^ AC Immune SA, Lausanne Switzerland

## Abstract

**Background:**

Quantitative systems pharmacology (QSP) models have emerged as useful tools for evaluating and understanding the efficacy of therapies for Alzheimer’s disease (AD). Bringing together a clinical focus with the mechanistic detail of systems biology modelling, QSP models are well‐suited to the complexity of AD and have been used to predict treatment outcomes and support regulatory submissions. Therapies targeting amyloid‐beta have dominated recent AD clinical development efforts culminating in the recent approvals of anti‐amyloid monoclonal antibodies.

**Method:**

To inform and facilitate future QSP model development, a systematic review of published QSP models focused on amyloid targeting AD therapies was completed. A search of the PubMed and Web of Science databases was conducted on September 5^th^, 2024. Search results were reviewed by a single reviewer at three levels: title screening, abstract screening, and full text screening. Predefined exclusion and inclusion criteria were applied to identify a final set of published QSP models for which reported model structure, development, and predictions were summarized. Shared and contrasting model features were identified across the included models. A set of model quality features was scored against a checklist adapted from recent ‘best practice' guidelines for QSP modelling.

**Result:**

Literature searches identified 494 candidate publications. Review and application of inclusion/exclusion criteria resulted in a total of nine distinct QSP model publications. All nine models were QSP models of AD used to simulate treatment effects for one or more anti‐amyloid therapy. Seven of these publications utilised compartmental models of amyloid aggregation and clearance, while two used network models of amyloid‐beta accumulation across the brain. Model quality scores were generally low and ranged from 6/15 to 9/15. Key quality issues related to model validation and reproducibility were identified, e.g., none of the nine papers provided access to executable model code.

**Conclusion:**

The assessment of nine QSP models provided a useful context to inform ongoing QSP model development and refinement efforts, such that future models may better inform therapeutic strategies for the treatment of AD. In particular, enhanced focus on quality issues will serve to improve the utility of future QSP models.

